# Supplementary material for: Virtual Screening-Based Study of Novel Anti-Cancer Drugs Targeting G-Quadruplex
Source: Pharmaceutics. 2023 May 5;15(5):1414. doi: 10.3390/pharmaceutics15051414 (PMC10222998; doi:10.3390/pharmaceutics15051414)
Supplement: Supplementary file 1 [file pharmaceutics-15-01414-s001.zip › pharmaceutics-2140309-supplementary.pdf]

Supporting Information

Table S1. The physical properties and similarity score of 23 screened compounds

Number	logP	MW	HBA	HBD	RC	PSA	Polar
1	0.32	365.4256	4	1	3	73.74	46.264
2	0.52	383.4161	4	1	3	73.74	46.173
3	2.16	432.8973	5	1	3	83.22	49.502
4	2.02	402.4823	3	1	3	89.09	47.695
5	-0.03	425.4776	6	1	3	92.2	51.208
6	2.41	414.4451	3	1	3	60.85	49.17
7	1.16	424.4895	5	1	3	79.31	52.461
8	1.67	384.856	3	1	3	60.85	47.652
9	1.81	371.3806	2	0	4	43.86	43.797
10	2.04	316.3948	3	0	3	49.85	37.681
11	2.62	425.5222	2	0	5	43.86	58.738
12	3.18	348.4382	2	0	4	40.62	46.298
13	1.89	358.7757	4	0	4	67.87	42.202
14	0.97	432.1071	3	1	3	60.85	37.428
15	2.36	351.2383	2	0	3	40.62	36
16	1.47	328.3376	3	0	3	58.64	40.278
17	1.06	379.4522	3	0	4	53.09	48.286
18	-0.66	317.3828	3	0	3	61.88	37.197
19	2.2	326.3648	2	0	3	49.41	41.476
20	2.02	286.3688	2	0	3	40.62	35.209
21	2.23	356.3907	3	0	3	58.64	43.948
22	0.51	392.451	3	1	4	72.96	49.086
23	1.22	368.4014	3	1	3	60.85	45.591

logP: oil-water partition coefficient.
MW: molecular weight.
HBA: hydrogen bonded acceptor.
HBD: hydrogen bonded donor.
RC: relative crystallinity
PSA: polar surface area

Table S2. Docking scores of 23 hit compounds.

Number	Docking Score			
	**143D**	**1KF1**	**2HY9**	**2JPZ**
1	-6.36921	-3.30817	-5.4937	-3.87285
2	-5.30124	-3.06062	-4.59782	-3.74024
3	-4.37155	-3.14798	-4.90768	-4.53299
4	-5.8222	-2.61149	-4.86061	-4.02361
5	-4.82827	-3.41171	-5.08112	-4.50055
6	-5.01954	-2.71853	-5.00908	-4.88085
7	-5.01123	-3.61745	-4.81507	-4.20793
8	-4.9374	-2.95308	-5.01893	-5.65552
9	-4.08517	-5.7094	-5.34572	-6.78632
10	-5.10363	-6.11016	-6.20308	-5.03704
11	-5.39837	-4.33044	-5.55276	-4.28201
12	-5.45704	-4.75493	-4.22932	-6.91298
13	-6.4635	-6.66536	-6.12421	-5.48711
14	-4.93495	-5.62584	-6.16354	-5.45613
15	-6.00265	-5.94935	-5.92312	-5.72202
16	-6.96146	-6.54753	-6.49837	-4.79471
17	-5.67672	-5.86457	-6.00714	-6.33375
18	-3.93663	-3.83226	-5.04324	-4.83038
19	-6.22413	-5.06335	-6.52012	-4.70422
20	-5.30844	-6.15113	-6.20905	-6.28896
21	-5.56251	-6.66644	-6.62432	-4.64252
22	-5.11953	-5.30395	-6.21182	-6.2523
23	-5.53678	-2.95099	-5.07523	-5.88785

Table S3. 3D similarity calculation scores of 23 hit compounds.

Number	Template	FeatureScore	ShapeScore	HybridScore
1	1	0.44	0.785	1.227
2	1	0.44	0.785	1.227
3	3	0.338	0.672	1.01
4	1	0.455	0.68	1.136
5	2	0.348	0.605	0.954
6	1	0.521	0.729	1.25
7	3	0.273	0.676	0.95
8	6	0.345	0.693	1.039
9	1	0.516	0.594	1.111
10	1	0.434	0.688	1.132
11	4	0.317	0.638	0.955
12	3	0.521	0.668	1.19
13	3	0.398	0.667	1.066
14	5	0.357	0.547	0.904
15	6	0.396	0.798	1.194
16	6	0.386	0.693	1.079
18	3	0.343	0.69	1.033
19	3	0.494	0.712	1.206
20	2	0.485	0.629	1.114
21	6	0.407	0.673	1.081
22	6	0.423	0.666	1.089
23	6	0.401	0.684	1.08

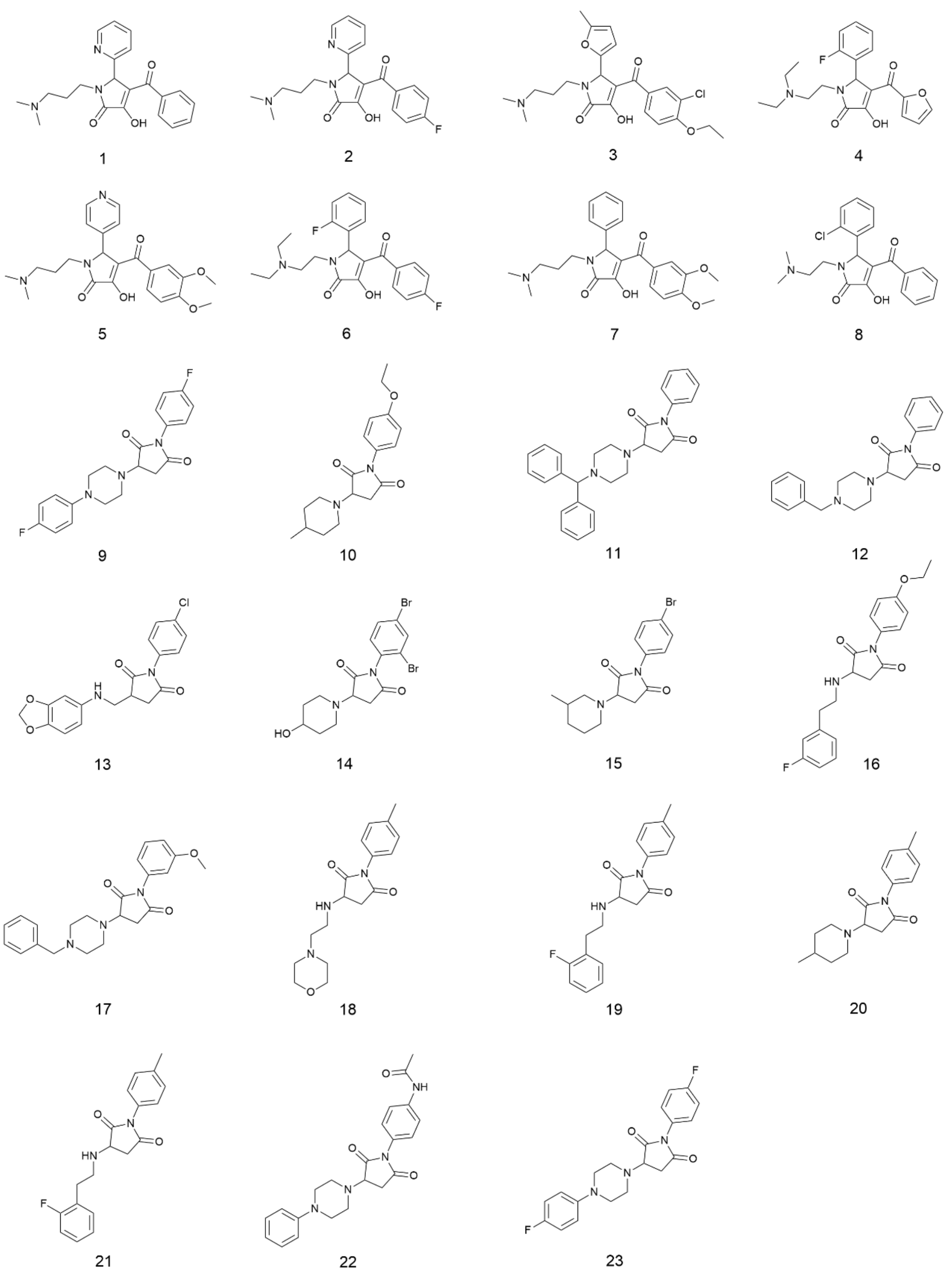

Figure S1. The chemical structures of 23 screened compounds.

Table S4. Names of 23 screened compounds

No.	Chemical Formula	Compound name
1	$C_{21}H_{23}N_3O_3$	4-benzoyl-1-[3-(dimethylamino)propyl]-3-hydroxy-5-(2-pyridinyl)-1,5-dihydro-2*H*-pyrrol-2-one
2	$C_{21}H_{22}FN_3O_3$	1-[3-(dimethylamino)propyl]-4-(4-fluorobenzoyl)-3-hydroxy-5-2(2-pyridinyl)-1,5-dihydro-2*H*-pyrrol-2-one
3	$C_{23}H_{27}ClN_2O_5$	4-(3-chloro-4-ethoxybenzoyl)-1-[2-(dimethylamino)ethyl]-3-hydroxy-5-(5-methyl-2-furyl)-1,5-dihydro-2H-pyrrol-2-one
4	$C_{21}H_{23}FN_2O_4$	1-[2-(diethylamino)ethyl]-5-(2-fluorophenyl)-3-hydroxy-4-(2-thienylcarbonyl)-1,5-dihydro-2H-pyrrol-2-one
5	$C_{23}H_{27}N_3O_5$	4-(3,4-dimethoxybenzoyl)-1-[3-(dimethylamino)propyl]-3-hydroxy-5-(4-pyridinyl)-1,5-di hydro-2H-pyrrol-2-one
6	$C_{23}H_{24}F_2N_2O_3$	1-[2-(diethylamino)ethyl]-4-(4-fluorobenzoyl)-5-(2-fluorophenyl)-3-hydroxy-1,5-dihydro-2H-pyrrol-2-one
7	$C_{24}H_{28}N_2O_5$	4-(3,4-dimethoxybenzoyl)-1-[3-(dimethylamino)propyl]-3-hydroxy-5-phenyl-1,5-dihydro-2H-pyrrol-2-one
8	$C_{21}H_{21}ClN_2O_3$	4-benzoyl-5-(2-chlorophenyl)-1-[2-(dimethylamino)ethyl]-3-hydroxy-1,5-dihydro-2H-pyrrol-2-one
9	$C_{20}H_{19}F_2N_3O_2$	1-(4-fluorophenyl)-3-[4-(4-fluorophenyl)-1-piperazinyl]-2,5-pyrrolidinedione
10	$C_{18}H_{24}N_2O_3$	1-(4-ethoxyphenyl)-3-(4-methyl-1-piperidinyl)-2,5-pyrrolidinedione
11	$C_{27}H_{27}N_3O_2$	3-(4-benzhydryl-1-piperazinyl)-1-phenyl-2,5-pyrrolidinedione
12	$C_{21}H_{23}N_3O_2$	3-(4-benzyl-1-piperidinyl)-1-phenyl-2,5-pyrrolidinedione
13	$C_{18}H_{15}ClN_2O_4$	3-[(1,3-benzodioxol-5-ylmethyl)amino]-1-(4-chlorophenyl)-2,5-pyrrolidinedione
14	$C_{15}H_{16}Br_2N_2O_3$	1-(2,4-dibromophenyl)-3-(4-hydroxy-1-piperidinyl)-2,5-pyrrolidinedione
15	$C_{16}H_{19}BrN_2O_2$	1-(4-bromophenyl)-3-(3-methyl-1-piperidinyl)-2,5-pyrrolidinedione
16	$C_{20}H_{21}FN_2O_3$	3-[(4-fluorobenzyl)amino]-1-(4-methoxyphenyl)-2,5-pyrrolidinedione
17	$C_{22}H_{25}N_3O_3$	3-(4-benzyl-1-piperazinyl)-1-(3-methoxyphenyl)-2,5-pyrrolidinedione
18	$C_{17}H_{23}N_3O_3$	1-(4-methylphenyl)-3-{[2-(4-morpholinyl)ethyl]amino}-2,5-pyrrolidinedione
19	$C_{19}H_{19}FN_2O_2$	3-{[2-(2-fluorophenyl)ethyl]amino}-1-(4-methylphenyl)-2,5-pyrrolidinedione
20	$C_{17}H_{22}N_2O_2$	1-(4-methylphenyl)-3-(4-methyl-1-piperidinyl)-2,5-pyrrolidinedione
21	$C_{19}H_{19}FN_2O_2$	1-(4-ethoxyphenyl)-3-{[2-(3-fluorophenyl)ethyl]amino}-2,5-pyrrolidinedione
22	$C_{22}H_{24}N_4O_3$	N-{4-[2,5-dioxo-3-(4-phenyl-1-piperazinyl)-1-pyrrolidinyl]phenyl}acetamide
23	$C_{20}H_{19}F_2N_3O_2$	1-(4-fluorophenyl)-3-(4-(4-fluorophenyl)piperazin-1-yl)pyrrolidine-2,5-dione

Compound 1

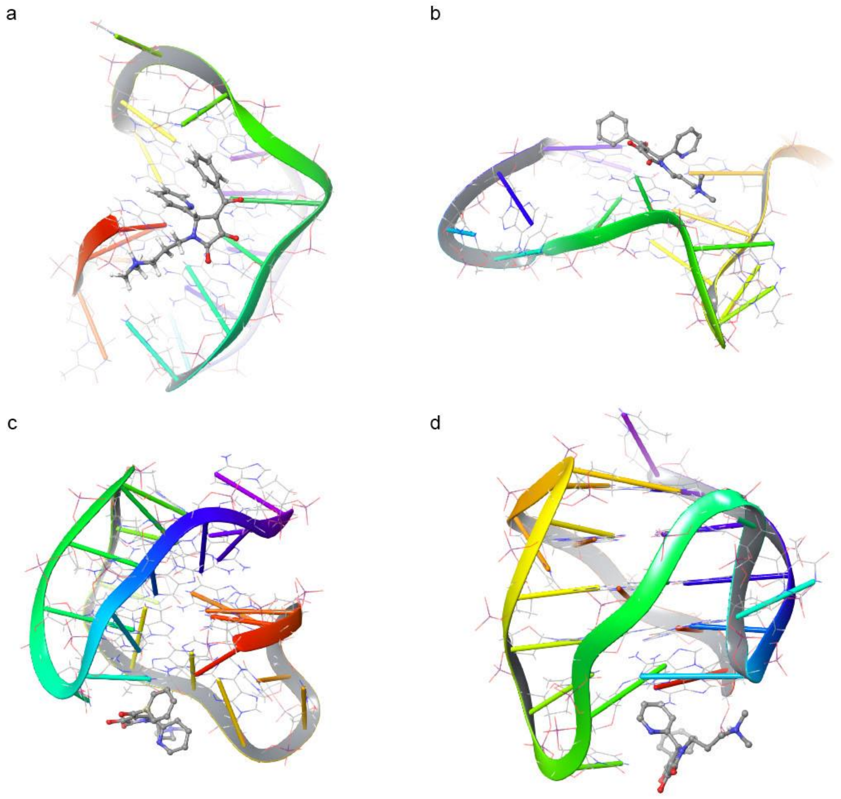

Compound 2

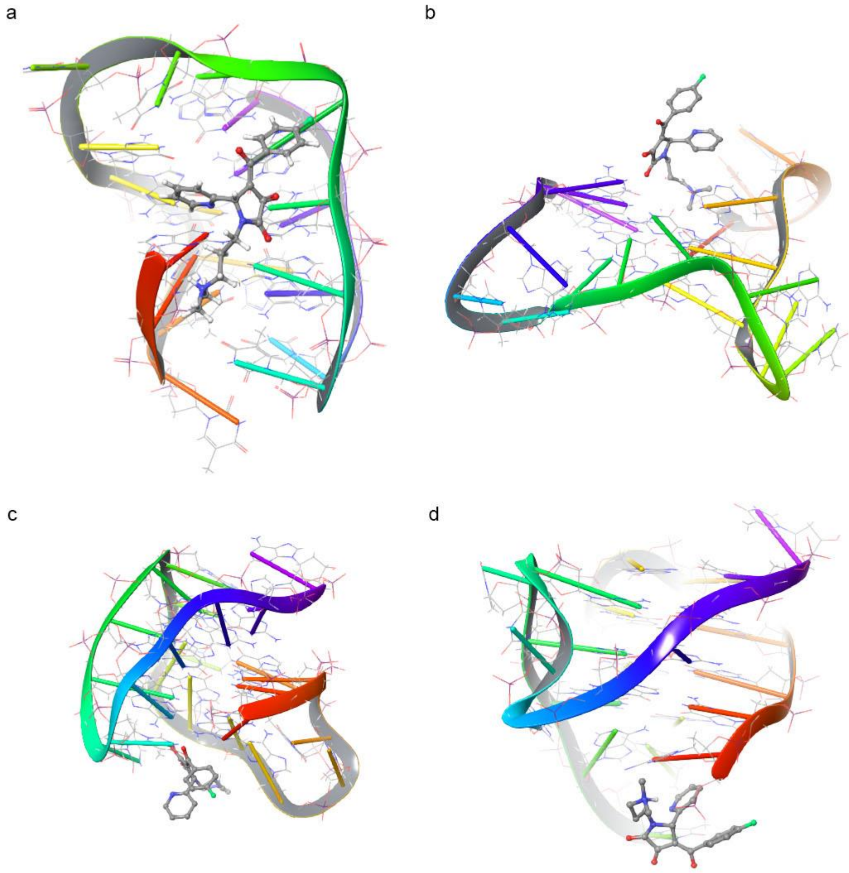

Compound 3

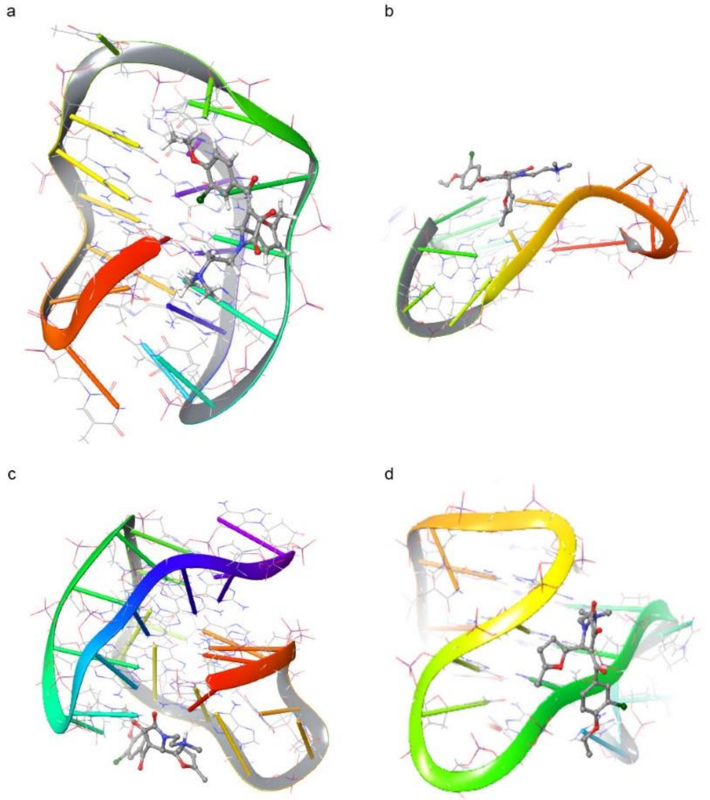

Compound 4

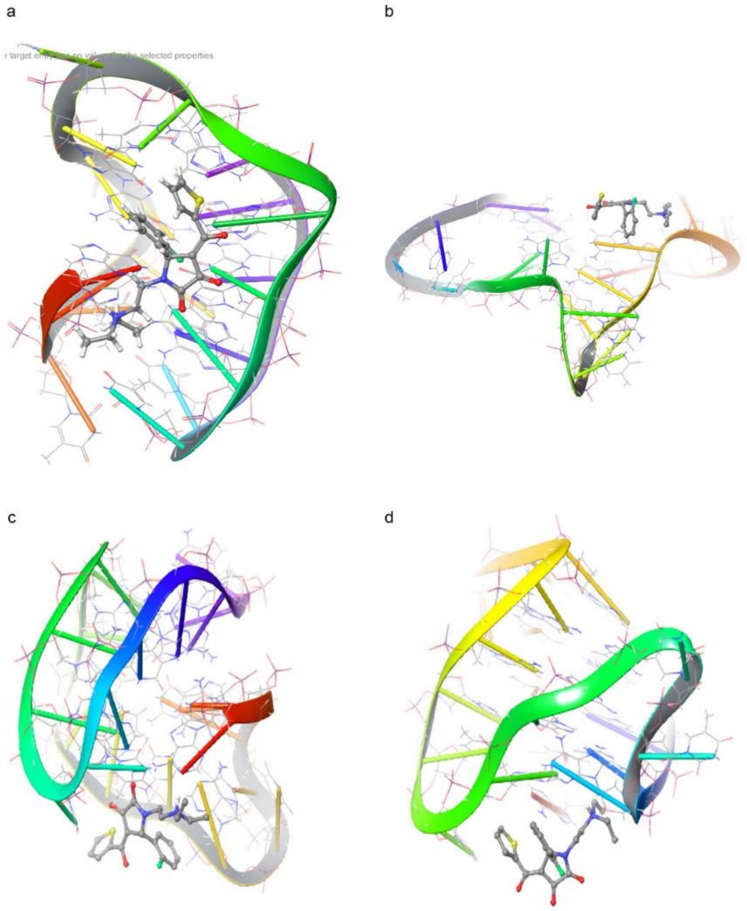

Compound 5

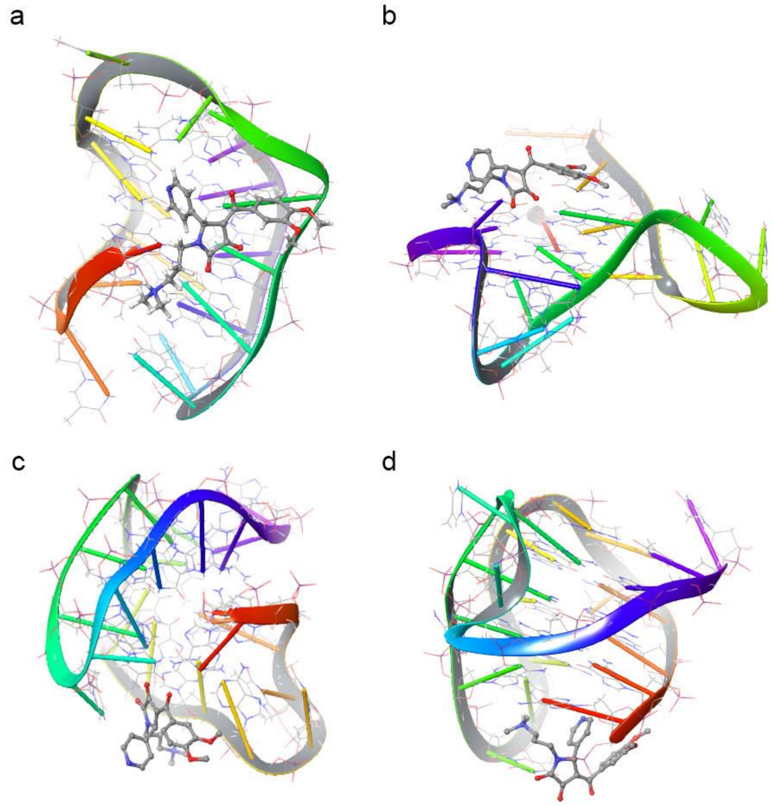

Compound 6

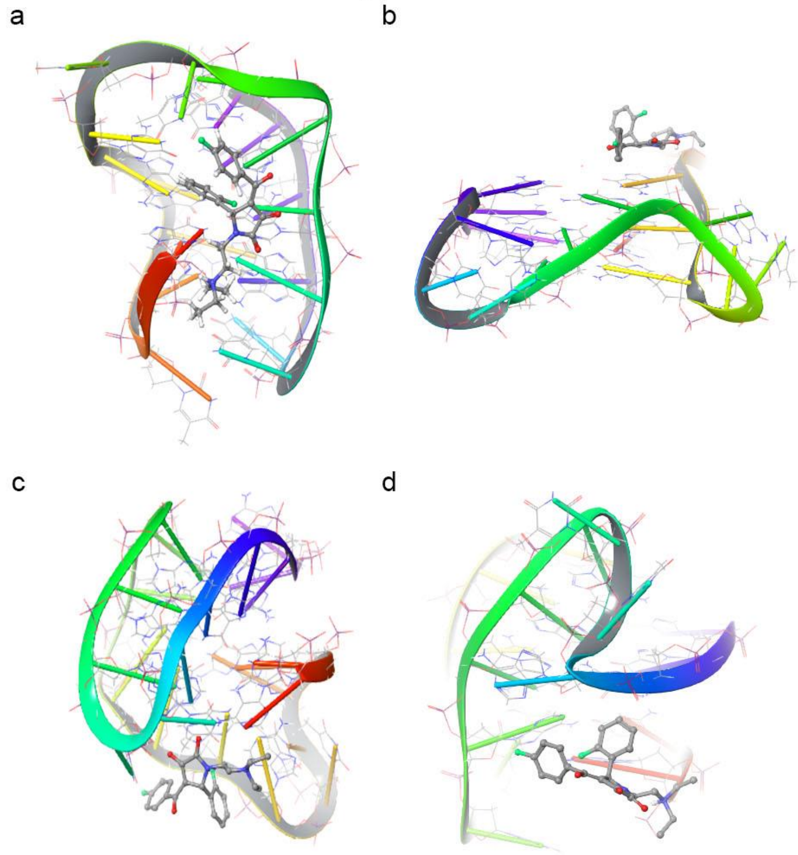

Compound 7

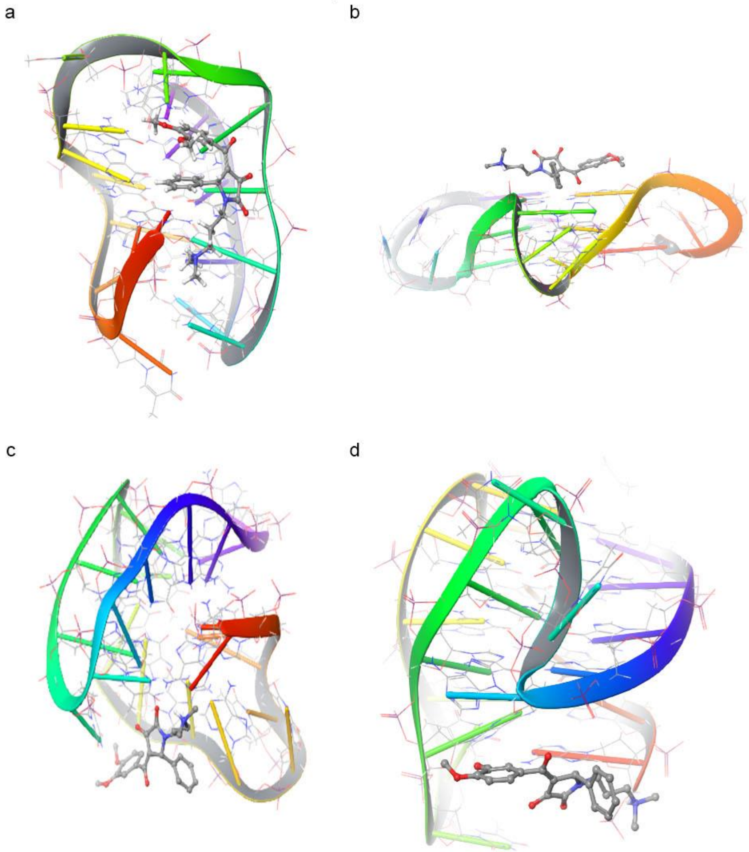

Compound 8

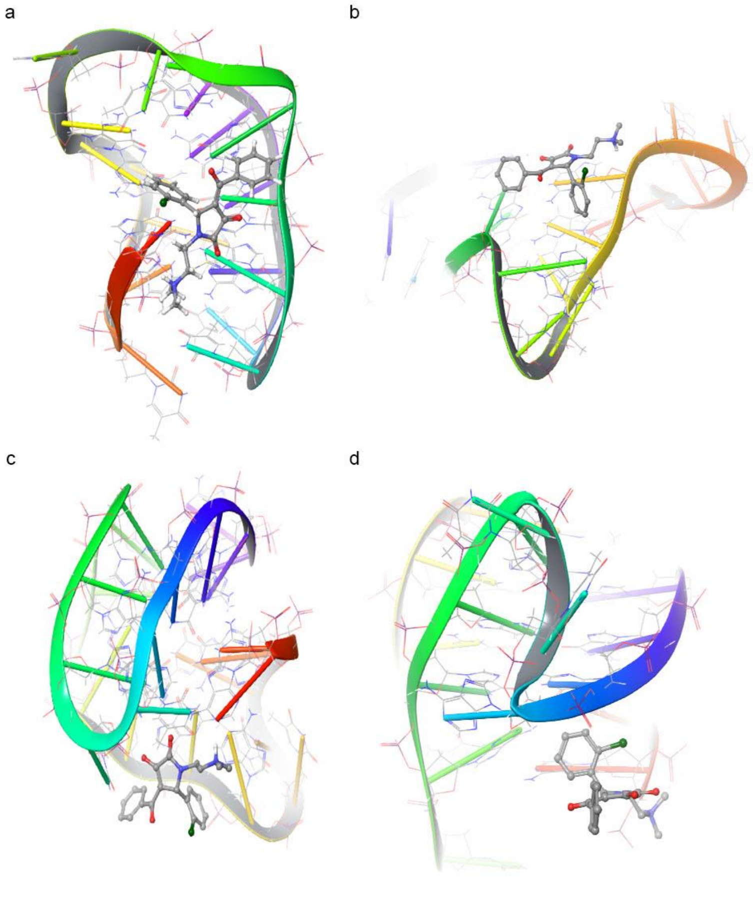

Compound 9

a

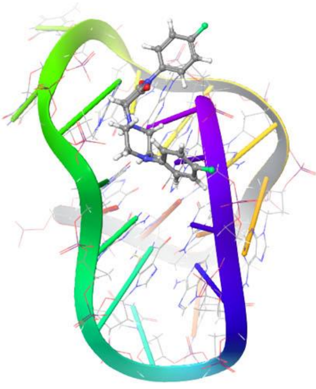

b

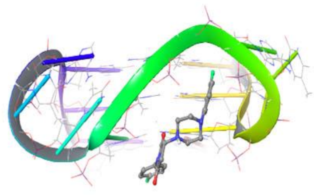

c

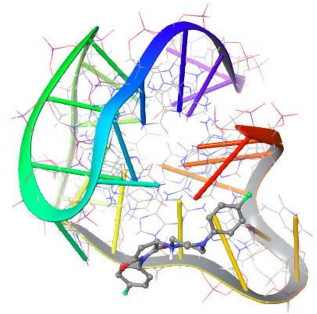

d

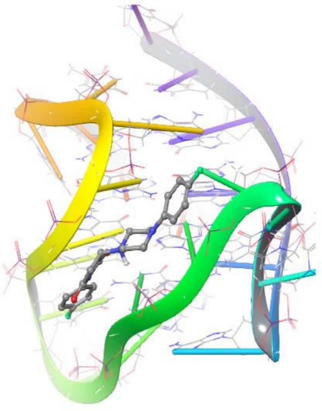

Compound 10

a

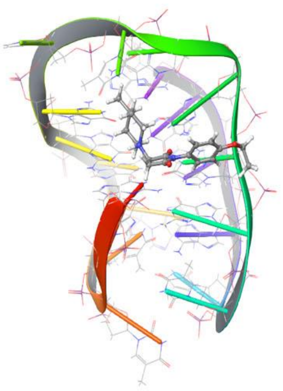

b

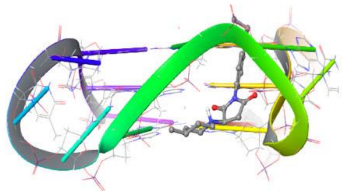

c

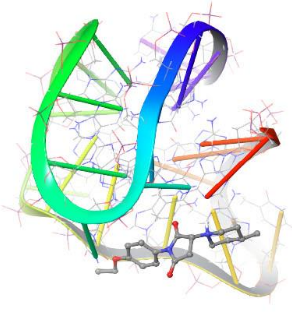

d

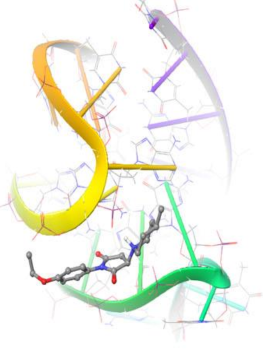

Compound 11

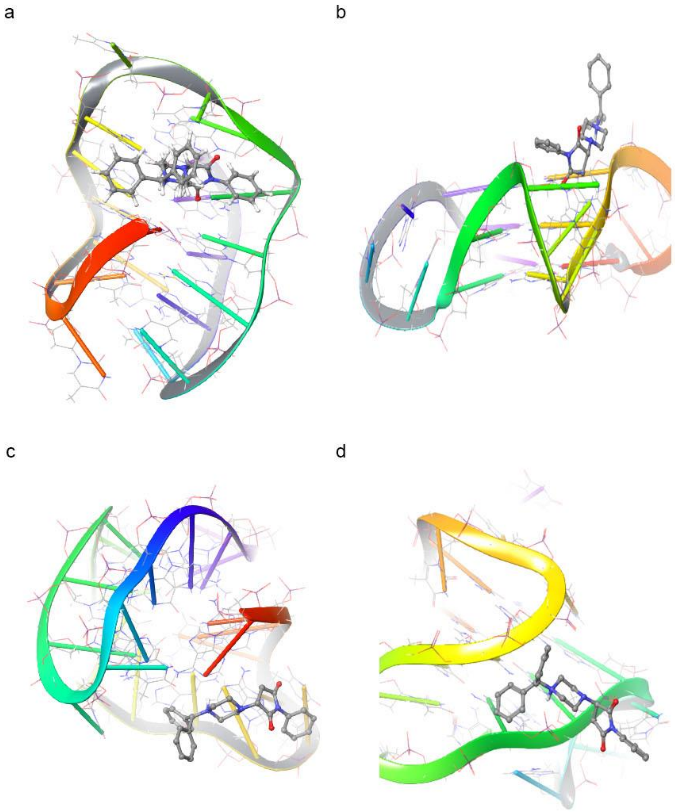

Compound 12

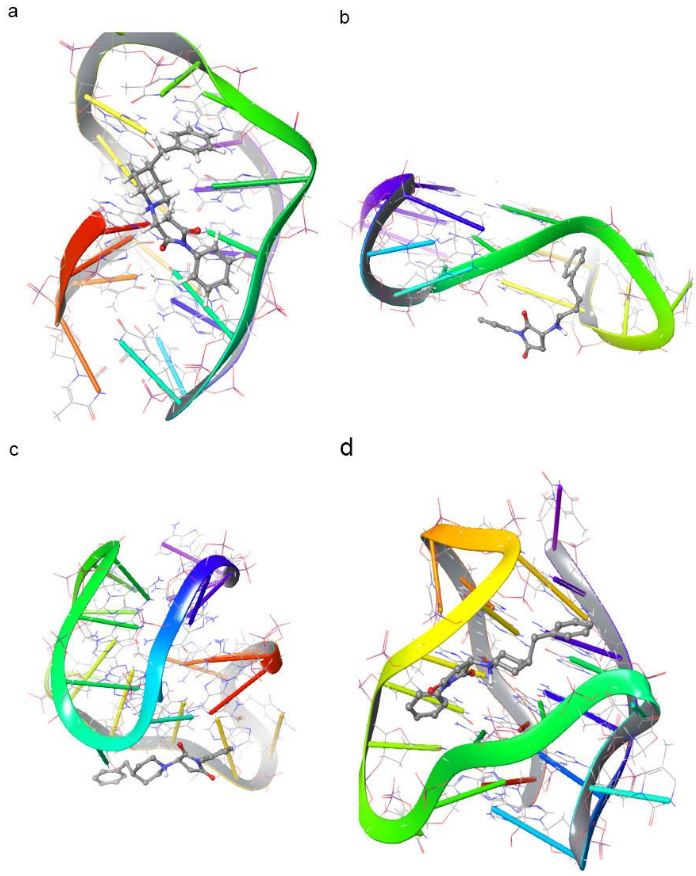

Compound 13

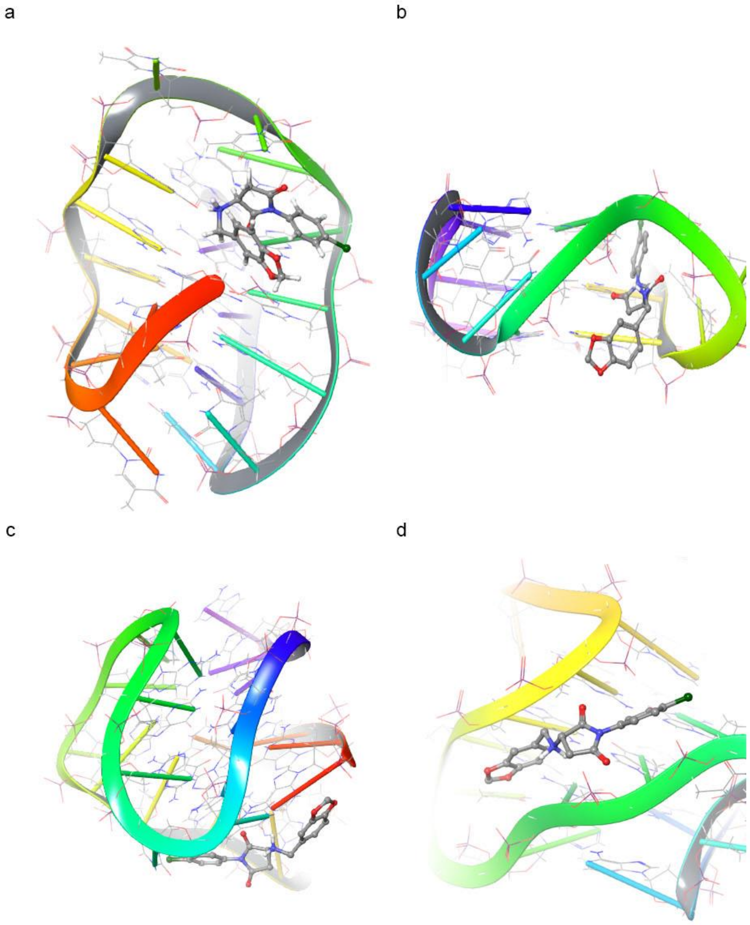

Compound 14

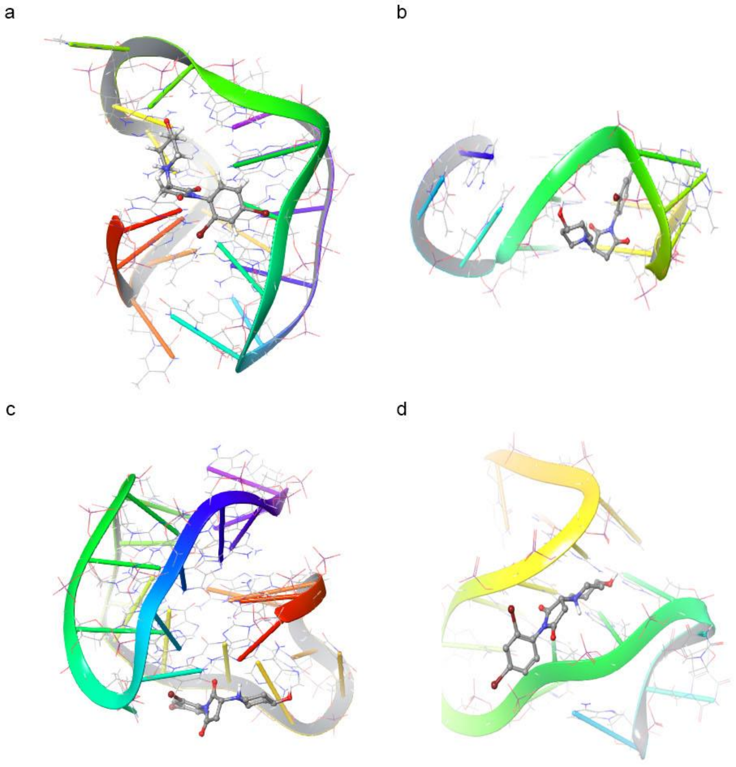

Compound 15

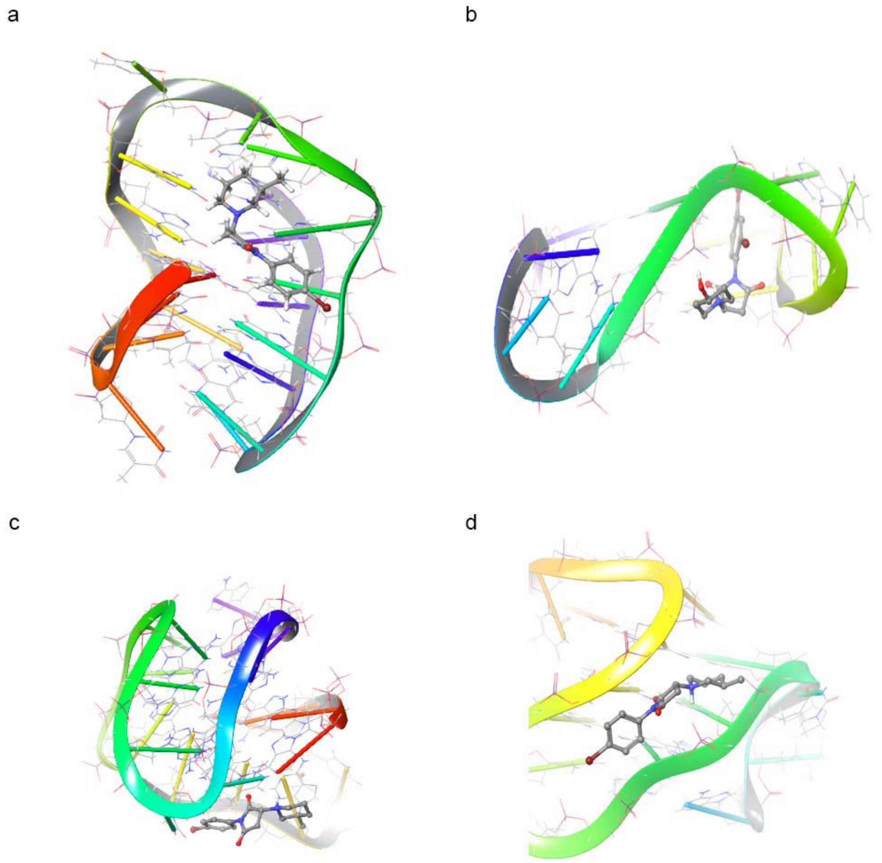

Compound 16

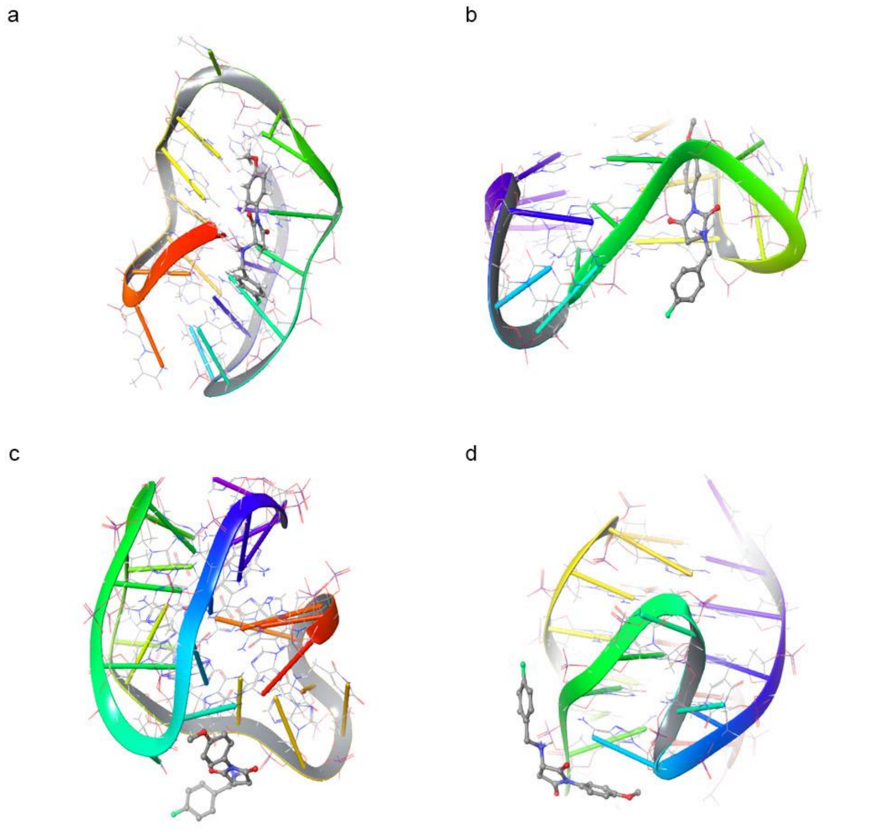

Compound 17

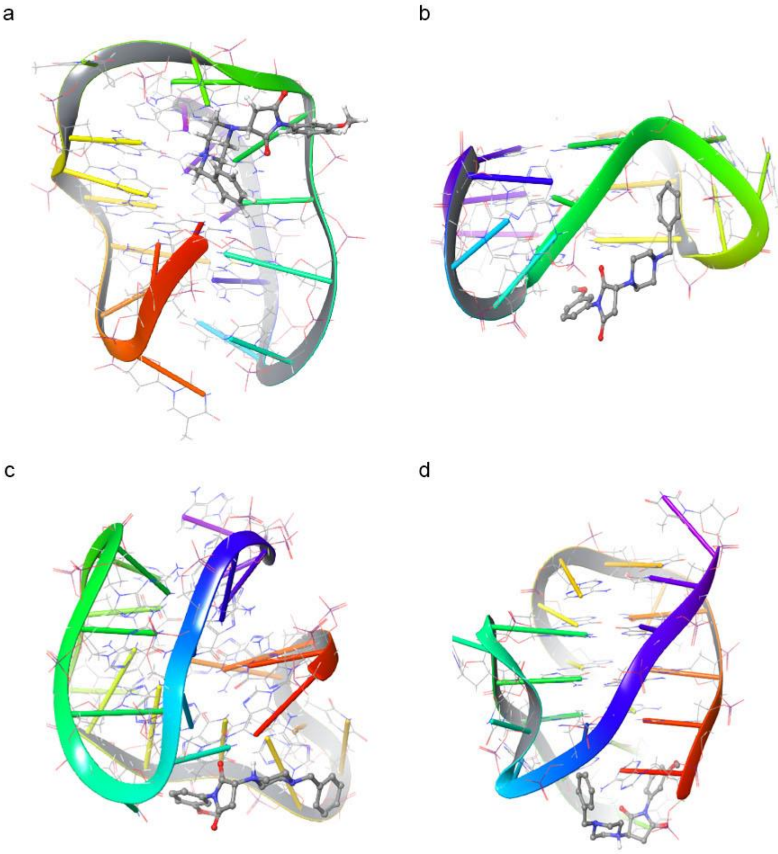

Compound 18

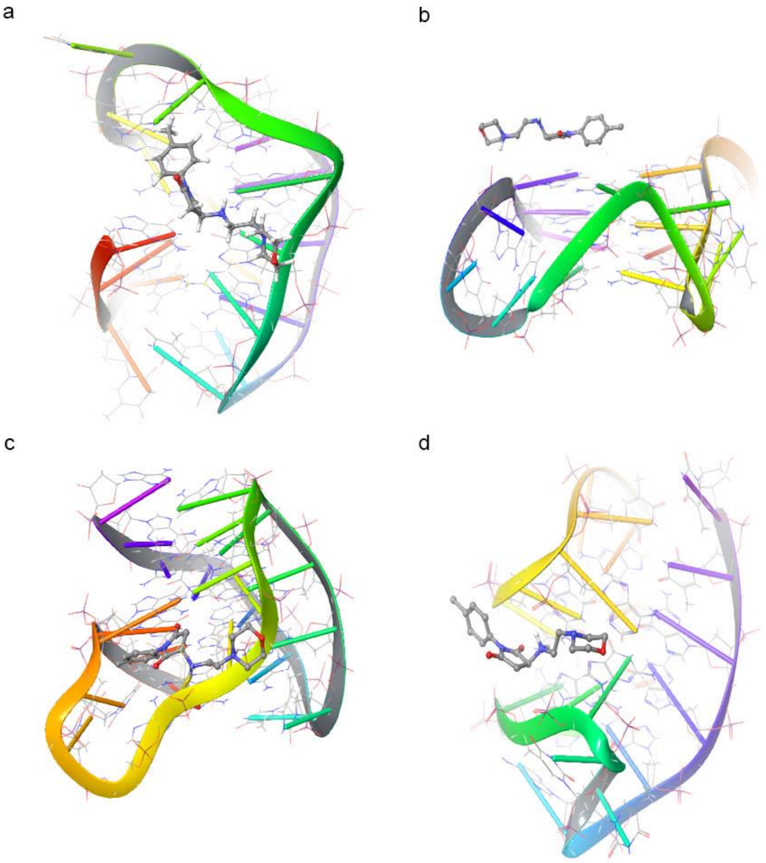

Compound 19

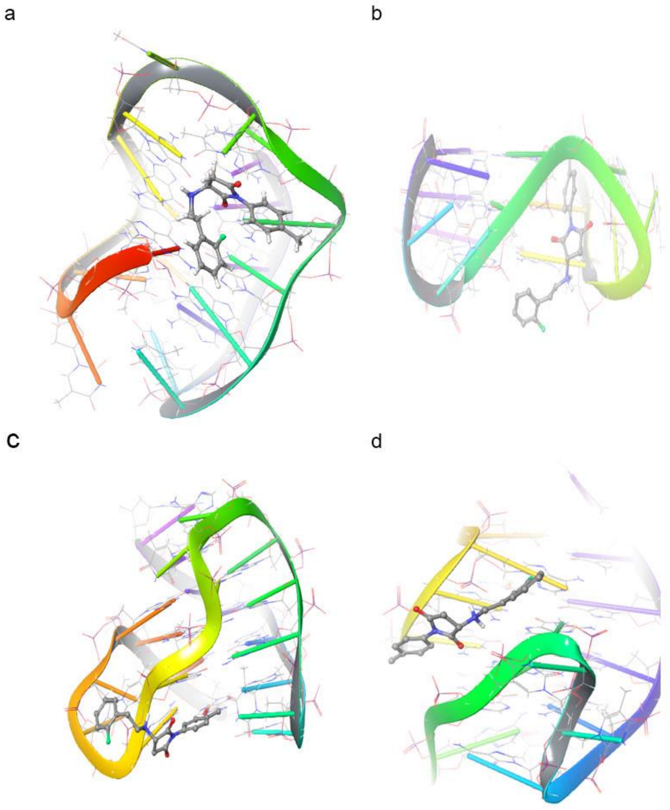

Compound 20

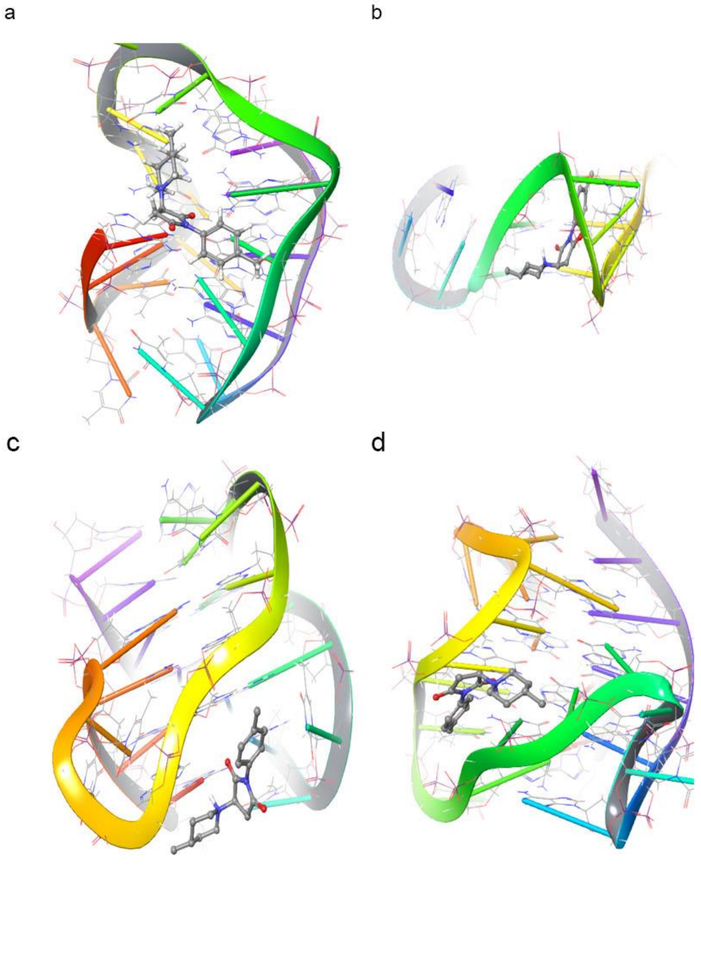

Compound 22

a

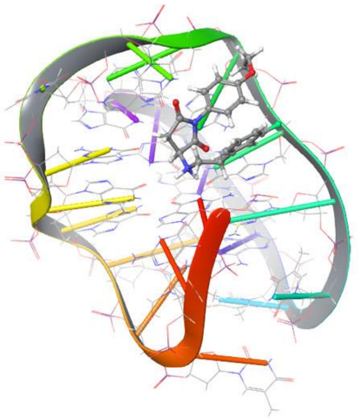

b

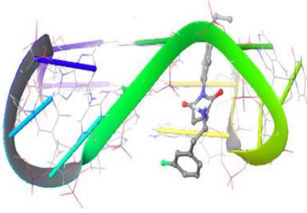

c

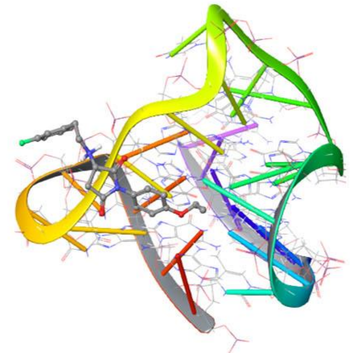

d

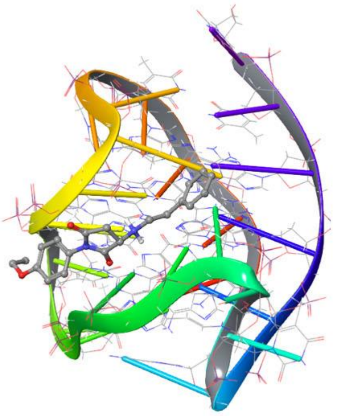

Compound 22

a

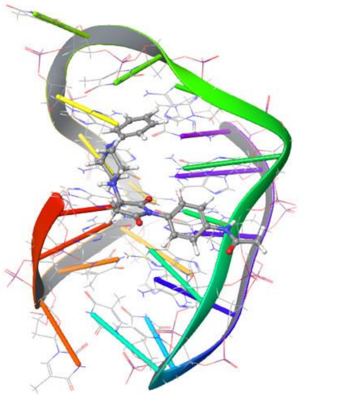

b

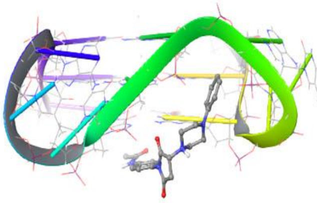

c

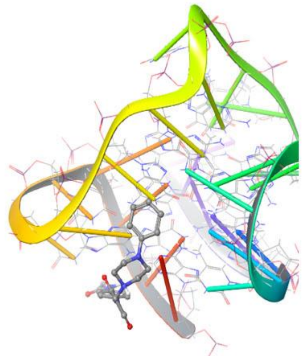

d

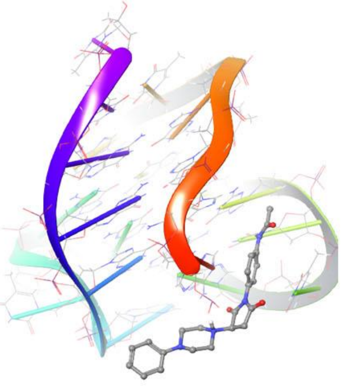

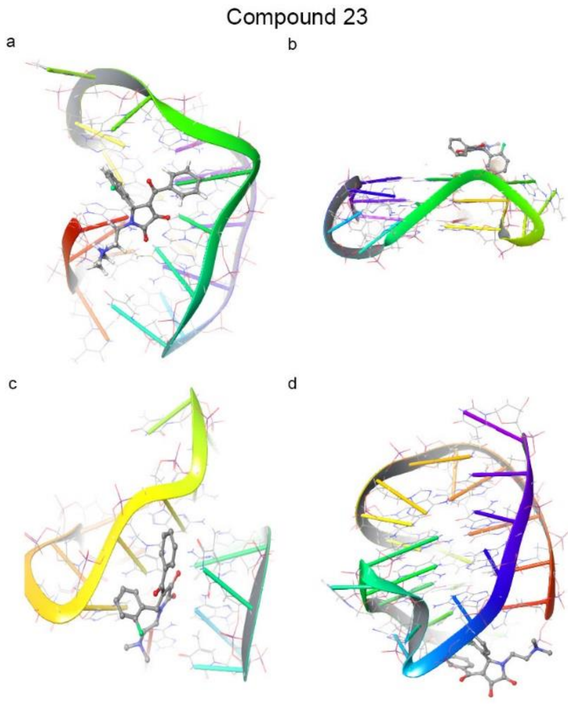

Figure S2. Four conformations of 23 screened compounds binding to G4 quadruplexes (a-b: 143D, 1KF1, 2HY9, 2JPZ) .
